# Buying efficiency: optimal hospital payment in the presence of double upcoding

**DOI:** 10.1186/s13561-019-0256-4

**Published:** 2019-12-28

**Authors:** Simon B. Spika, Peter Zweifel

**Affiliations:** 1 Department of Economics, University of Konstanz, Box 135, Konstanz, 78457 Germany; 2 Department of Economics, Emeritus, University of Zürich, Zürich, Switzerland

**Keywords:** Hospital organziation, Upcoding, Hierarchical principal-agent model, Nash bargaining model, Distribution of power

## Abstract

**Background:**

With DRG payments, hospitals can game the system by ’upcoding’ true patient’s severity of illness. This paper takes into account that upcoding can be performed by the chief physician and hospital management, with the extent of the distortion depending on hospital’s internal decision-making process. The internal decision making can be of the principal-agent type with the management as the principal and the chief physician as the agent, but the chief physicians may be able to engage in negotiations with management resulting in a bargaining solution.

**Results:**

In case of the principal-agent mechanism, the distortion due to upcoding is shown to accumulate, whereas in the bargaining case it is avoided at the level of the chief physician.

**Conclusion:**

In the presence of upcoding it may be appropriate for the sponsor to design a payment system that fosters bargaining to avoid additional distortions even if this requires extra funding.

## Introduction

Ever since the introduction of DRG payment of hospitals, there have been concerns about the truthfulness of their reporting. Because hospitals establish severity of illness, they are suspected by their sponsors to game the system by exaggerating true severity in an attempt to optimize revenue, by so-called ’upcoding’. Several empirical findings substantiate this suspicion ([14], [3], [2], [6]).

Upcoding strategies result in reimbursement that is higher than required for efficiency, and the sponsor of hospital services therefore needs information on whether and to what extent upcoding occurs in order to take appropriate countermeasures. Indeed, DRG payment is frequently supplemented by monitoring and sanctions that apply when false or biased reporting is detected. But since monitoring and imposing fines are not without their own cost, the optimal combination of payment, monitoring, and fining becomes an issue ([7]). To address it, however, an analysis of hospitals’ reporting strategy is called for.

An important fact is that upcoding can occur at two points along the flow of information in a typical hospital with a central management and several clinical departments ([6]). True severity is only observed by the clinical department which conducts diagnostics and treatment. This information is forwarded to the coding division of management as a medical record. There, diagnoses and treatments are encoded according to standardized classification systems such as ICD-10. The encoded information is fed into a special software that uses an algorithm to assign a DRG to the case. This DRG is reported to the sponsor (the health insurer or the government) who effects payment accordingly. Clearly, a first opportunity for upcoding exists in the clinical departments by overstating the severity of illness as documented in the medical record. One example is understating birth weight because low birth weight indicates high severity (for empirical evidence, see [6]). Another example is prolonging stays in the intensive-care unit without medical necessity. The second upcoding opportunity exists in the management’s coding division, which has some leeway in determining the main diagnosis or in the interpretation of medical reports, enabling the encoding of additional diagnoses or treatments. As a consequence, reporting to the sponsor may be the result of an accumulation of distortions at both levels of the hospital’s hierarchy.

For upcoding to occur, there must be incentives inducing the individual in charge to misrepresent the severity of illness. Yet with DRG payment in place, such incentives clearly exist for management since a DRG with a higher case-weight increases hospital revenue. As to the clinical department, it may benefit from overstating severity as well, provided net revenue generated drives internal resource allocation (e.g. because a budget target must be achieved). This type of incentive is typical for DRG payment, and it is often intensified by benchmarking mechanisms which use the cost/casemix ratio as a performance indicator.

However, the internal incentives depend on other organizational features of the hospital as well. In particular, departmental involvement in the setting of the formal and informal rules coordinating management and clinical departments is of importance. Typically, there is a formal separation between the allocation and use of resources. The authority to determine the internal resource allocation through budgeting is vested with central management, while chief physicians decide on the use of these resources for treating patients subject to a budget constraint. This mechanism is of the principal-agent type, with management acting as the principal and the head of a clinical department acting as the agent. An important feature of such a mechanism is that both players maximize their own utility, without taking the effect of their actions on the joint surplus into account. Theory predicts that in the presence of asymmetry of information and divergent objectives of the two players, this leads to distortions due to information rents and a solution that usually is not Pareto-efficient ([9] ch. 1).

Commonly, however, a hospital’s budgeting process in fact involves its departments. In a first round, central management communicates a target to the department. The department, having detailed information on demand and medical technology, then suggests adjustments of the target. While central management has the final say, this process provides the chief physician directing the department with a measure of influence. After all, chief physicians combine medical and management skills often with a high degree of assertiveness and perseverance in the pursuit of their objectives. The result is a negotiation with management allowing them to reach a higher utility than attainable by simply accepting targets imposed top-down. Hence, this budgeting process in fact suggests a bargaining solution which reflects the chief physician’s bargaining power relative to that of the management. In contrast to the principal-agent mechanism, with bargaining the players are more likely take into account how joint surplus is affected by their actions. Thus, with a bargaining mechanism, the internal distortion due to asymmetry of information may be internalized, resulting in a Pareto-optimal outcome.

For the sponsor, the difference between the two types of internal decision-making is relevant. Since in the presence of a principal-agent mechanism the information advantage of the chief physician is likely to result in a distortion away from a Pareto-efficient solution, the incentive on the side of hospital management to exploit its informational advantage over the sponsor by biasing its reports gives rise to an accumulation of distortions. This accumulation is well known in contract theory, where the implications of information processing have been discussed in connection with the delegation of authority in internal hierarchies ([11]). Specifically, [10] show that information passing through multiple levels of a hierarchy may result in information rents accruing at each level of the hierarchy, causing a cumulative loss of efficiency. A bargaining mechanism that avoids this accumulation therefore would be preferable from the sponsor’s perspective.

These considerations suggest that a theoretical analysis of hospital payment should not only reflect the internal asymmetry of information regarding the severity of cases but also the decision-making mechanism involving management and chief physician in the specification of the hospital’s objective function which governs a hospital’s response to the incentives provided by the payment system. [1] do examine the effect of an internal asymmetry of information, but with management and chief physician interacting in a principal-agent relationship only. Management decides on physician payment and high-tech treatment capacity, while the chief physician decides on the number of patients treated using this capacity. True case-mix is only observed by the chief physician, while hospital management can only associate case-mix with a high or low DRG value. At the top of the hierarchy, all the sponsor knows is the probability distribution of the case-mix. The utility-maximizing chief physician converts his/her informational advantage into information rent, causing an additional distortion away from the optimal allocation as seen by the sponsor, compared with a situation of no internal asymmetry of information.

In turn, different decision-making mechanisms are considered by [4]. The authors distinguish between a bargaining and a principal-agent mechanism to find that the internal decision-making mechanism matters for hospital behavior. In particular, management and chief physicians maximize joint surplus in the negotiation alternative regardless of the distribution of bargaining power. In the principal-agent setting, the two players make their decisions simultaneously in a Cournot game, failing to take the implications of their decisions on joint surplus into account. While this seems to speak in favor of the bargaining alternative, Gallizzi and Miraldo show that if case-mix is private information of the hospital and capital cost of high-tech treatment is not excessive, the sponsor fares better with the principal-agent alternative. The reason is that with the regulatory instruments assumed to be at its disposal, the sponsor is able to suppress the hospital’s information rent in this case.

The novelty of this paper is to combine features of the contributions by [1] and [4]. Its core objective is to relate a hospital’s reporting strategy to the presence of internal asymmetry of information between management and chief physician as well as to the balance of power between these two players and to demonstrate the relevance of both elements for the design of the optimal payment scheme by the sponsor. It adds to the existing literature in three ways. First, it combines internal asymmetry of information and decision making-mechanism in analyzing hospital behavior. Second, it highlights the relevance of the balance of power within a hospital. Third, it provides guidance in the design of a payment system that not only provides optimal incentives for a given internal structure of the hospital but also fosters an internal decision-making mechanism that benefits the sponsor.

The structure of this paper is as follows. In “Methods” section, the model and its assumptions are presented. “Results” section contains the analysis of the model. In “Full internal information” section, the outcomes without internal asymmetry of information are outlined. Next, in “Internal asymmetry of information” section, the two decision-making mechanisms are analyzed for the case of internal asymmetry of information, permitting the chief physician to report truthfully only if this is in her/his interest. “Discussion” section contains a discussion of the model and its implications, “Conclusions” section concludes the paper.

## Methods

This section is devoted to the specification of the model in terms of patients types, objectives and participation constraints, the flow of information, contracts between the players, the two internal decision making mechanisms and the timeline.

### Patients and treatment

Let a sponsor (a government agency or social health insurer) delegate the treatment of a patient with a certain illness to a hospital that has a monopoly in its catchment area. Case severity of the patient is represented by a one-dimensional parameter *θ*, which is distributed with cumulative distribution function *F*(*θ*) on the interval $\Theta = [ \underline {\theta },\overline {\theta } ]$. This distribution is common knowledge and satisfies the monotone hazard property, i.e. $h(\theta) = \frac {1-F(\theta)}{f(\theta)}$ is strictly decreasing. Furthermore convexity is assumed, *h*^′^(*θ*)<0,*h*^″^(*θ*)≥0, which is valid for common distributions like the (symmetrically truncated) normal or the uniform distribution. Medical treatment consists of a single service of quantity *q*. For simplicity fixed costs are set to zero and the price for one unit of *q* is one. Thus treatment cost equal *q*^1^. The sponsor observes and reimburses realized treatment cost *q*.

### Objectives and participation constraints

The relevant decision makers in the hospital are the management (M) and a chief physician (CP). M is responsible for financial solvency, while treatment is planned and conducted by the CP according to her or his professional autonomy. CP’s total utility per patient treated is given by

1 $$  U = \theta V(q) + t^{I}.  $$

The term *θV*(*q*) in CP’s objective function captures CP’s intrinsic motivation and may be interpreted as CP’s valuation of the treatment as such, expressed in monetary units. It is assumed continuous, strictly increasing and concave, reflecting a beneficial effect of treatment with decreasing marginal utility^2^. The severity of illness acts as a multiplier. This can be justified by noting that patients benefit more from treatment when their illness is severe. Alternatively, one may argue that the CP derives utility from treating a patient of high severity, demonstrating her or his skills. The CP further derives utility from an internal transfer per patient treated *t*^*I*^, effected by M. This transfer does not affect CP’s personal income, which is assumed to be determined exogenously. The transfer *t*^*I*^ could be interpreted as an additional budget the CP can use for activities in the department (e.g. to finance the participation in congresses). As to the valuation of *t*^*I*^, risk neutrality is assumed, reflecting the fact that the transfer is mainly used for financing fringe benefits accruing to co-workers in the department (who therefore are affected by its variation). Finally, to accept a patient for treatment, the CP must attain a minimum reservation utility. To simplify the analysis, it is assumed that denial of treatment is without further consequences and the CP can do some other work yielding an exogenously given utility equal to zero. Thus, the CP accepts the patient for treatment only if^3^

2 $$  U\geq 0.  $$

Management is assumed to focus exclusively on financial matters^4^. Since the cost *q* is covered by the sponsor, M’s objective is to maximize

3 $$  P = t^{E} - t^{I},  $$

with *P* symbolizing profit per patient, which amounts to the difference between an (external) transfer *t*^*E*^ received from the sponsor per patient treated and the payment to the CP. In the present context, for M to agree to the treatment of a patient in the hospital, the outcome must result in a non-negative profit calling for the *ex-post* participation constraint^5^

4 $$  P \geq 0.  $$

At the top of the hierarchy, the sponsor aims at maximizing patient utility net of expenditure,

5 $$  W = \theta B(q) - t^{E} - q.  $$

Here, *W* symbolizes welfare per patient and *θB*(*q*) the patient’s gross benefit scaled according to severity *θ*, reflecting the assumption that the sponsor prefers a severely ill patient to be treated over a moderately ill one^6^. *B*(*q*) is continuous, strictly increasing, and concave.

### Flow of information

At the time a patient presents herself at the hospital, the CP conducts a costless first examination to determine severity *θ*. Hence, actual realizations of *θ* can only be observed by the CP. Next, the chief physician reports $\hat {\theta }^{I} \in \Theta $ to M (internal report). On the top of the hierarchy, the sponsor communicates with M only and cannot observe CP’s report $\hat {\theta }^{I}$. Rather, the sponsor only observes a report $\hat {\theta }^{E} \in \Theta $ made by M (external report), who acts as the intermediary between the sponsor and CP.

### Contracts

The sponsor designs and offers to M a payment scheme which consists of the external transfer to the hospital and a cost target per patient treated, both depending on severity of illness as reported by M, $\left \{t^{E}(\hat {\theta }^{E}), q^{E}(\hat {\theta }^{E})\right \}\; \forall \hat {\theta }^{E} \in \Theta $.

Within the hospital, a contract defines the internal transfer to the CP and an internal cost target the CP must abide to when treating a patient. The internal transfer and the internal cost target both depend on the CP’s report of case severity, $\{t^{I}(\hat {\theta }^{I}), q^{I}(\hat {\theta }^{I})\}\; \forall \hat {\theta }^{I} \in \Theta $. However, since the external cost target $q^{E}(\hat {\theta }^{E})$ must be met by the hospital, the external report $\hat {\theta }^{E}$ implicitly defines the internal cost target. Therefore, the internal contract is equivalent to a contract defining the internal transfer and M’s reporting strategy depending on the CP’s internal report, i.e. $\{t^{I}(\hat {\theta }^{I}), \hat {\theta }^{E}(\hat {\theta }^{I})\}\; \forall \hat {\theta }^{I} \in \Theta $.

### Internal decision-making mechanisms

The hospital’s formal statutes define internal decision making with M having the authority to set the initial offer $\{t^{I}(\hat {\theta }^{I}), \hat {\theta }^{E}(\hat {\theta }^{I})\}$. This mechanism is of the principal-agent (PA) type, with M acting as the principal and the CP as the agent. However, by rejecting the initial offer, the CP can initiate a bargaining process involving M and CP that results in the contract $\{t^{I}(\hat {\theta }^{I}), \hat {\theta }^{E}(\hat {\theta }^{I})\}$. In keeping with the standard approach of economic theory, the outcome of this bargaining process is the solution of a Nash bargaining game, in which the CP’s share of the surplus increases with her or his bargaining power relative to M. In the following, CP’s relative bargaining power is symbolized by *w*∈(0,1), while that of M is (1−*w*). In contrast to CP’s reservation utility stated in Eq. (2), *w* captures personal characteristics which determine her/his bargaining behavior, i.e. self-confidence, aggressiveness, and stamina. M’s relative bargaining power (1−*w*) in turn reflects the position of the administration in the power structure of the hospital.

### Timeline of the model

The timeline of the model comprises four stages:

1. The sponsor offers the contract {*t*^*E*^(*θ*),*q*^*E*^(*θ*)} ∀*θ*∈*Θ* to M.
2. M determines the reporting strategy $\hat {\theta }^{E}(\hat {\theta }^{I})$ and payment $t^{I}(\hat {\theta }^{I})$ for every possible report of severity by the CP at stage No. 4 and offers this contract to the CP.
3. CP decides whether or not to engage in negotiations. If she/he engages in negotiations, the reporting strategy and payment determined by M in stage No. 3 are replaced by a bargaining solution for $\hat {\theta }^{E}(\hat {\theta }^{I})$ and $t^{I}(\hat {\theta }^{I})$.
4. A patient seeks treatment at the hospital. The CP observes case severity *θ* and decides on treatment. If treatment is denied, the game ends. If the patient is admitted, the CP reports $\hat {\theta }^{I}$ to M and CP provides the quantity $q^{I}(\hat {\theta }^{I})$ of service. M reports $\hat {\theta }^{E}(\hat {\theta }^{I})$ according to the strategy determined at stage No. 2 (stage No. 3 respectively) and the payments are made.

## Results

### Full internal information

As a benchmark assume the CP always reports truthfully^7^. With the bargaining solution, the CP and M aim at maximizing their respective share of their expected joint surplus in negotiating the internal transfer and the external report for every case severity in a Nash bargaining process. Note that joint surplus *S*(*θ*)=*P*(*θ*)+*U*(*θ*) is independent of internal payment *t*^*I*^(*θ*) and that the unrestricted Nash bargaining solution is Pareto-efficient (see e.g. [12] ch. 2). Because the bargaining solution in fact is not impaired by distortions due to asymmetric information if the CP reports always truthfully, this implies that the bargaining solution entails an external reporting $\hat {\theta }^{E}(\theta)$ that maximizes joint surplus for every case severity independently of CP’s bargaining power *w*,

6 $$  \max_{\hat{\theta}^{E}}S(\theta) = t^{E}(\hat{\theta}^{E}) + \theta V(q^{E}(\hat{\theta}^{E}))\;\; \forall \theta \in \Theta,  $$

while the internal payment *t*^*I*^(*θ*) is used to split surplus according to the relative bargaining power. In the following, denote the solution to (6) as *efficient* reporting and the maximized surplus as *S*_*eff*_(*θ*).

With the PA mechanism, an identical solution results. With the PA setting, M seeks to maximize the external transfer $t^{E}(\hat {\theta }^{E})$ received from the sponsor while keeping the internal transfer *t*^*I*^(*θ*) as low as possible. This is equivalent to the maximization of joint surplus while keeping the CP’s utility at the lowest possible level. As in the case with bargaining, reporting is used to maximize joint surplus, i.e. in the PA setting, external reporting is efficient as well and maximizes (6). Further, since the PA solution must yield at least the same utility for the CP as the bargaining solution (otherwise the PA mechanism is replaced by bargaining), the internal transfer has the same value in the two settings.

For the design of the optimal contract by the sponsor, $\{t^{E}(\hat {\theta }^{E}), q^{E}(\hat {\theta }^{E})\}$, all relevant information is contained in Eq. (6). However, the sponsor must account for the fact that severity of illness is only observed within the hospital. As noted in the Introduction, payment systems are often combined with monitoring and sanctioning by the sponsor in order to mitigate the effect of information asymmetry. But if these options are not available (as it is assumed in this analysis), the revelation principle implies that no contract performs better than a direct mechanism {*t*^*E*^(*θ*),*q*^*E*^(*θ*)} which induces truthful reporting, i.e. $\hat {\theta }^{E}(\theta)= \theta $. To achieve truthful reporting, the hospital’s incentive compatibility constraint must be satisfied, which requires surplus to increase with case severity, taking account of Eq. (6). In addition, to ensure that the contract is accepted for every case severity, M’s and CP’s joint surplus must be non-negative at the lowest level of case severity. With these two constraints, the optimal contract yields a surplus of zero for the hospital in the case of lowest severity, while the optimal cost ceiling *q*^*E*^(*θ*) achievable for the sponsor is given by

7 $$  \theta B_{q} (q^{E}(\theta)) + (\theta -h(\theta)) V_{q}(q^{E}(\theta)) = 1.  $$

Note that (7) implies that the optimal cost ceiling is strictly increasing with case severity, i.e. $\frac {dq^{E}(\theta)}{d\theta }>0$. The optimal allocation is characterized by the standard rent extraction-efficiency trade-off (see e.g. [8] ch. 1). Since $h(\overline {\theta }) = 0$, the cost target and the treatment quantity exhausting the cost ceiling are efficient only for a patient with the highest severity level, for whom marginal benefit equals marginal expenditure. But at the same time, the hospital’s undesirable information rent is maximum for such a patient, since incentive compatibility requires the surplus to increase with severity. Conversely, the information rent decreases with severity until it reaches zero at the minimum severity level $\underline {\theta }$, where surplus equals zero. Finally, the distortion of *q*^*E*^(*θ*) from its efficient value increases with *θ* because at a low severity, the inverse hazard rate is high (by assumption, *h*^′^(*θ*)<0). In the following, the optimal contract without internal asymmetry of information is referred to as $\left \{t_{eff}^{E}(\theta), q_{eff}^{E}(\theta)\right \}$.

### Internal asymmetry of information

Now, the assumption that the CP always issues a truthful report is relaxed. The game is solved using backward induction starting at stage No. 4.

#### CP’s information rent

At stage No. 4, the CP observes severity of illness *θ*, decides on whether to treat the patient or not and issues the report $\hat {\theta }^{I}(\theta)$ to M. Given the internal contract $\{t^{I}(\theta), \hat {\theta }^{E}(\theta)\}$, the CP’s maximization problem at this stage reads

8 $$  \max_{\hat{\theta}^{I}} U(\theta) = t^{I}(\hat{\theta}^{I}) + \theta V(q^{E}(\hat{\theta}^{E}(\hat{\theta}^{I}))).  $$

An exaggerated CP’s report would correspond to the second variant of upcoding (see the Introduction again). [6] point out that upcoding by the clinical department can hardly be detected, justifying the assumption that monitoring is not worthwhile for M. Instead, the focus is on an internal contract that results in the best allocation achievable without monitoring. By the revelation principle, it is sufficient to analyze a direct mechanism that induces the CP to report truthfully. Incentive compatibility can be established by applying the envelope theorem to (8) at $\hat {\theta }^{I}(\theta) = \theta $,

9 $$  \dot{U}(\theta) = V(q^{E}(\hat{\theta}^{E}(\theta))) > 0,  $$

implying that for a truthful report at stage No. 4, CP’s utility needs to increase with case severity. In addition the CP’s participation constraint *U*(*θ*)≥0 ∀*θ*∈*Θ* must be satisfied, which in combination with (9) guarantees the CP a utility amounting at least to

10 $$ R(\theta) = \int^{\theta}_{\underline{\theta}}V(q^{E}(\hat{\theta}^{E}(\tilde{\theta}))) d\tilde{\theta},  $$

where *R*(*θ*) denotes CP’s information rent. Since $R(\underline {\theta })=0$ and $R(\theta)>0\;\forall \; \theta > \underline {\theta }$, the expected information rent *E*[*R*(*θ*)] is strictly positive. Note that this information rent accrues to the CP independently of her/his bargaining power *w*.

##### **Conclusion 1**

In the case of internal asymmetry of information, the CP attains an expected utility at least equal to the expected information rent *E*[*R*(*θ*)]>0.

#### The bargaining solution

At stage No. 3 the CP decides on whether to accept M’s offer or to engage in bargaining. Assume that he/she opts for bargaining. In this case, *t*^*I*^(*θ*) and $\hat {\theta }^{E}(\theta)$ are negotiated between the two players for every severity *θ*, given the contract {*t*^*E*^(*θ*),*q*^*E*^(*θ*)} offered by the sponsor to M at stage No. 1. As without internal asymmetry of information, the players aim to maximize their share of expected surplus. The Nash bargaining outcome in terms of optimal payment and reporting is the solution to the maximization problem

11 $$ \begin{aligned} && \pi(\theta) = \left(E\left[t^{E}(\hat{\theta}^{E}(\theta)) - t^{I}(\theta)\right] \right)^{1-w} \left(E\left[t^{I}(\theta) + \theta V(q^{E}[\hat{\theta}^{E}(\theta)])\right] \right)^{w},  \end{aligned}  $$

but the solution now must not only satisfy M’s and CP’s participation constraints, but also induce CP to report true severity in stage No. 4. Thus, by the revelation principle the best attainable solution not only satisfies M’s and CP’s participation constraints but also CP’s incentive compatibility constraint (9)^8^

As shown in “CP’s information rent” section, the CP’s participation and incentive compatibility constraints together require that the solution yield at least a non-negative utility in the guise of an information rent. However, given that the joint surplus exceeds CP’s information rent, there is a minimum bargaining power for which the CP’s bargained share of surplus exceeds her/his information rent. If this is the case, CP’s and M’s participation constraints are not binding. This in turn creates leeway for structuring the internal payment *t*^*I*^(*θ*) such that CP’s incentive compatibility constraint is satisfied without impairing the maximization of joint surplus. In that event, efficient reporting $\hat {\theta }^{E}(\theta)$ is also the solution to the bargaining problem despite internal asymmetry of information. For a proof, see Appendix A, where it is also shown that CP’s bargaining power must be equal ore above the threshold value

12 $$  \bar{w} := \frac{E[R_{eff}]}{E[S_{eff}]},  $$

where *E*[*R*_*eff*_] denotes the CP’s expected information rent with efficient reporting.

##### **Conclusion 2**

If CP’s bargaining power *w* is equal or above $\bar {w}:= \frac {E[R_{eff}]}{E[S_{eff}]}$, the bargaining solution yields efficient reporting and maximizes joint surplus even in the case of asymmetry of information.

#### The solution to the PA mechanism

For the analysis of the internal contract designed by M at stage No. 2, assume for the moment that the CP cannot engage in bargaining at the next stage No. 3. Through its choice of the internal payment and the reporting strategy, M seeks to maximize expected profit given the payment system selected by the sponsor,

13 $$  \max_{t^{I}(\theta), \hat{\theta}^{E}(\theta)} E[P(\theta)] = E[ t^{E}(\hat{\theta}^{E}(\theta))-t^{I}(\theta)],  $$

subject to CP’s participation and incentive compatibility constraints. Since M dislikes leaving any surplus to the CP, the internal transfer *t*^*I*^(*θ*) is optimally structured such that the CP just attains the information rent defined in (10), implying that CP’s participation constraint binds, hence $U(\underline {\theta }) =R(\underline {\theta })= 0.$ With these constraints, the optimal reporting strategy is the solution to (see Appendix A)

14 $$ \begin{aligned}\max_{\hat{\theta}^{E}(\theta)} \tilde{P}(\theta)= t^{E}(\hat{\theta}^{E}) + \theta V(q^{E}(\hat{\theta}^{E}(\theta))) - h(\theta) V(q^{E}(\hat{\theta}^{E}(\theta))) \;\forall \theta \in \Theta. \end{aligned}  $$

In contrast to the bargaining case analyzed in the previous section, the binding participation constraint in the case of the PA mechanism causes distortions away from the optimal reporting strategy since M needs to trade off efficiency against the elicitation of CP’s information rent. In Eq. (14) the distortions are reflected by $h(\theta) V(q^{E}(\hat {\theta }^{E}(\theta)))>0$ which can be interpreted as cost incurred by M for ’buying’ a truthful report from the CP ([10]). Note that because the reporting strategy is not efficient anymore, CP’s information rent now is below the information rent with efficient reporting. In the following, CP’s information rent given the PA mechanism is denoted by *R*_*PA*_(*θ*).

##### **Conclusion 3**

In the case of internal asymmetry of information, the PA mechanism yields a distorted hospital reporting strategy.

#### Equilibrium mechanism with internal asymmetry of information

Given the solutions of the bargaining and the PA mechanisms analyzed in “The bargaining solution” and “The solution to the PA mechanism” sections respectively, which one constitutes the equilibrium mechanism and what does this imply for the hospital’s behavior?

It turns out that for $w \geq \bar {w} = \frac {E[R_{eff}]}{E[S_{eff}]}$, the CP and M both prefer bargaining while for $w < \bar {w}$, the PA setting is optimal for both players. The intuition is that for $w \geq \bar {w}$, CP’s bargained share of efficient surplus exceeds her/his information rent associated with the PA setting, while for $w < \bar {w}$, it is not worthwhile for the CP to engage in bargaining, since her/his bargained share is too low. For M on the other hand, the PA contract defined in “The solution to the PA mechanism” section implies the optimal trade-off between efficiency and rent elicitation if $w < \bar {w}$. If however $w \geq \bar {w}$ holds, M could possibly dissuade the CP from bargaining by paying a sufficiently high lump sum in addition to the PA contract. But this is not optimal because the payment needed to compensate the CP exceeds M’s loss due to bargaining. Therefore, M optimally accepts bargaining if $w \geq \bar {w}$. The detailed argumentation is provided in Appendix A.

##### **Conclusion 4**

In the case of internal asymmetry of information, bargaining occurs if $w \geq \bar {w}$. The hospital’s reporting strategy is efficient and maximizes joint surplus. If on the other hand $w < \bar {w}$, the PA mechanism prevails, causing the reporting strategy to be distorted and maximizing (14).

#### Optimal payment system with internal asymmetry of information - Buying efficiency

In the view of Conclusion 4, it may be advantageous for the sponsor to make an extra payment to induce bargaining within the hospital; in this way, the sponsor would ’buy efficiency’. This idea is pursued below.

Assume first that the CP accepts the PA contract, while the hospital adopts the reporting strategy defined by (14). If M is to pass on CP’s report without manipulation, the incentive compatibility constraint (derived from applying the envelope theorem to (14)),

15 $$  \dot{\tilde{P}}(\theta) = (1 - h'(\theta)) V(q^{E}(\theta)) > 0,  $$

must be satisfied to induce $\hat {\theta }^{E}(\theta)= \theta $. As derived in Appendix A, the optimal allocation the sponsor can achieve when the PA mechanism is relevant, is characterized by a joint surplus for CP and M which increases with severity but is zero at the lowest severity, and a first-order condition for the cost ceiling that reads

16 $$  \theta B_{q}(q^{E}(\theta)) +\left(\theta - h(\theta)(2 - h'(\theta)\right)V_{q}(q^{E}(\theta)) = 1.\\  $$

Denote the optimal payment system to achieve the optimal allocation as $\{t^{E}_{PA}(\theta), q^{E}_{PA}(\theta)\}$. By juxtaposing (16) with (7), an additional distortion becomes evident. The reason is that in the case of the PA setting combined with internal asymmetry of information, M’s report is already distorted, causing the distortions across the two levels of the hierarchy to accumulate. This is to the detriment of the sponsor, who now must pay twice to obtain truthful reports (see [11]). As in the case with no internal asymmetric information, the cost ceiling *q*^*E*^(*θ*) is increasing and efficient only at the highest severity level. With asymmetric information added, inefficiency extends downward to all $\theta <\overline {\theta }$. In sum, the sponsor’s trade-off between efficiency and rent extraction deteriorates under the PA setting combined with internal asymmetric information compared with the case without internal information asymmetry.

Recall that in the case of the bargaining alternative in contrast, the hospital’s reporting strategy $\hat {\theta }^{E}(\theta)$ is efficient and the additional distortion is avoided. Therefore, the sponsor essentially prefers bargaining. However, efficiency comes with a price when internal asymmetry of information is relevant. Even if the reporting strategy with bargaining is efficient regardless of internal asymmetry of information, the sponsor cannot employ the payment system $\left \{t_{eff}^{E}(\theta), q_{eff}^{E}(\theta)\right \}$ defined in “Full internal information” section. This system is designed to leave the hospital just the information rent it can obtain with efficient reporting as surplus, i.e. *E*[*S*_*eff*_]=*E*[*R*_*eff*_]. Yet in the view of (12) bargaining does not occur as the equilibrium mechanism, since *w*>1 is impossible.

But the sponsor can ’buy’ efficient reporting. Since the threshold $\bar {w}$ decreases with expected surplus, the sponsor could augment the payment $t_{eff}^{E}(\theta)$ by a lump-sum payment *ε*>0 in order to increase joint surplus *E*[*S*_*eff*_] without affecting CP’s information rent *E*[*R*_*eff*_]. Specifically, the sponsor can increase joint surplus up to a level where the threshold $\bar {w}$ equals CP’s actual bargaining power *w*. By proposition 1 in Appendix A, the lump sum needed to achieve $\bar {w}=w$ must satisfy the condition

17 $$ \epsilon(w) = \frac{1-w}{w}E[R_{eff}].  $$

The lump sum *ε*(*w*) approaches zero as *w*→1 (CP has all bargaining power), but it is decreasing and strictly convex in *w* and reaches infinity for *w*→0 (CP has no bargaining power). On the other hand, because the distortions associated with the PA mechanism are to the detriment to the sponsor, the sponsor’s willingness to pay for replacing it is positive. Therefore there exists an *ε*(*w*)>0 which makes the sponsor indifferent between the internal PA and the bargaining setting. This value in turn determines a unique threshold for CP’s bargaining power $\bar {w}^{*}$. These considerations lead to the following rule for the choice of the optimal payment system depending on CP’s bargaining power,

18 $$ \begin{aligned}  \{t^{E^{*}}(\theta), q^{E^{*}}(\theta)\} = \left\{\begin{array}{ll} \left\{t^{E}_{PA}(\theta), q^{E}_{PA}(\theta)\right\} &\text{if} \qquad w < \bar{w}^{*}\\ \left\{t^{E}_{eff}(\theta)+\epsilon(w), q^{E}_{eff}(\theta)\right\}&\text{if} \qquad w \geq \bar{w}^{*}. \end{array}\right. \end{aligned}  $$

If $w < \bar {w}^{*}$, the sponsor optimally employs the PA alternative $\left \{t^{E}_{PA}(\theta), q^{E}_{PA}(\theta)\right \}$ to achieve the allocation associated with (16). For $w \geq \bar {w}^{*}$, however, it is optimal for the sponsor to pay the lump sum *ε*(*w*) in addition to the external contract $\left \{t^{E}_{eff}(\theta), q^{E}_{eff}(\theta) \right \}$ to foster internal bargaining.

##### **Conclusion 5**

The additional distortions associated with the PA mechanism are to the detriment of the sponsor. To avoid these distortions, the sponsor can foster bargaining by augmenting the payment $t_{eff}^{E}(\theta)$ by a lump-sum payment *ε*(*w*). Specifically, it is worthwhile for the sponsor to ’buy’ efficiency if CP’s bargaining power *w* is equal or above the threshold $\bar {w}^{*}$.

## Discussion

Upcoding is an issue affecting all hospital payment systems that offer a higher reimbursement for more severe cases. Upcoding can occur at two points along the flow of information in a typical hospital. True case severity is only observed by the clinical department establishing the diagnosis and rendering treatment, giving rise also to internal asymmetry of information to the detriment of hospital management. The clinical department may overstate the severity of illness in its report to management e.g. aiming to benefit from a more generous resource allocation. The second opportunity for upcoding obtains for management vis-à-vis the sponsor, by overstating the case severity reported by the clinical department in an attempt to increase payment by the sponsor. These distortions may accumulate along the flow of information, thwarting the sponsor’s quest for efficiency and cost containment.

This paper analyzes a hierarchical model, with the chief physician (CP) at the bottom and the sponsor at the top of the hierarchy and management (M) acting as the intermediary between sponsor and CP. It combines the internal information asymmetry with the decision-making mechanism coordinating CP and M to derive the impact on the hospital’s reporting strategy and hence attainment of the sponsor’s objective. Two alternatives are considered, a principal-agent setting where M confronts the CP with a take-it-or-leave-it offer, and Nash bargaining - provided the CP is willing to engage in a negotiation with M. Without internal asymmetry of information, both mechanisms are shown to result in a Pareto-efficient allocation between M and CP and identical hospital reporting strategies. If internal asymmetry of information is present, however, the principal-agent mechanism leads to an accumulation of information rents reaped by CP and M, which causes the sponsor to incur an additional efficiency loss. In contrast, the bargaining alternative has the potential to re-establish Pareto efficiency between CP and M by avoiding these distortions. The condition is that the CP’s bargaining power is equal or above a minimum threshold level, allowing him or her to appropriate a sufficiently high share of the joint surplus to absorb the CP’s information rent and implying that his or her participation constraint is not binding.

The model thus demonstrates that in the presence of internal asymmetry of information, the CP’s relative bargaining power is an important determinant of the hospital’s reported case severity and hence the sponsor’s optimal choice of hospital payment. In addition, since the threshold level for CP’s bargaining power decreases with CP’s and M’s joint surplus, it may be appropriate for the sponsor not only to design the optimal payment system in response to the prevailing internal decision-making mechanism, but to pay an extra lump sum designed to encourage the CP to engage in bargaining, thus avoiding distortions induced by the principal-agent mechanism.

This work is subject to several limitations. Most importantly, risk neutrality of both CP and M concerning the internal transfer between them is assumed for simplicity. Given risk neutrality, available surplus can be reallocated between M and CP without affecting its total value, resulting in efficient reporting. Notably, risk aversion on the side of the CP would cause the marginal utility of the internal transfer to decrease, thus affecting the joint surplus in utility terms. However, this consideration does not affect the crucial insights of this analysis. Also with a risk averse CP, it is still the case that a CP with bargaining power equal or above a certain threshold obtains a utility from negotiation which exceeds his or her information rent, a sufficient condition to prevent the accumulation of rents.

Usually, the sponsor cannot observe the CPs’ relative bargaining power within the hospital. However, ownership could be a pertinent indicator. Privately owned, for-profit hospitals are typically part of a group with clear business objectives to be pursued in the interest of private stakeholders. Here M is likely to be the dominating player endowed with bargaining power exceeding that of the CP, suggesting a principal-agent mechanism is in place. By way of contrast, in public hospitals the balance of power tends to be more in favor of the CP. One reason is diffuse objectives (e.g. provision of sufficient health care, excellence in medical research). Another reason may be that the traditional position of the CP as the ’fixed star’ in a hospital’s universe is still prominent in a public hospital. Consequently, the bargaining solution is the more plausible assumption for public hospitals than for privately owned ones. Ceteris paribus, the model thus predicts more intense upcoding behavior in private than in public hospitals.

This prediction is supported to some extent by empirical studies. [14] analyzed U.S. Medicare claims data for hospital discharges with DRGs related to respiratory infections. They measured upcoding using the ratio of discharges with the DRG triggering the highest expected reimbursement relative to those with a DRG from the set of all DRGs related to respiratory diseases. The upcoding rates in for-profit hospitals were up to 70% higher than those in public hospitals. Summarizing their findings, the authors state, ’...we view upcoding as symptomatic of how for-profit and not-for-profit hospitals differ in managerial behavior and the organizational balance of power inside the hospital’. The model presented in this paper provides theoretical support for their view.

More recently, [5] tested for upcoding in the market for neonatal intensive care. Using German data, they compared the distribution of reported birth weights of newborns with the distribution that was to be expected in the absence of financial incentives. Financial incentives to misreport birth weight arise since the German DRG-based reimbursement system defines eight thresholds in birthweight below which expected payment increases substantially. The authors found that upcoding rates were higher in counties with only for-profit perinatal centers than in counties with only public centers. The difference, however, was not statistically significant.

## Conclusions

The hospital’s internal power structure is likely to be a determinant of its internal decision making, which in turn needs to be considered for explaining and predicting hospital behavior in response to financial incentives. Thus, the sponsor’s optimal choice of payment scheme is found to depend on the hospital’s internal power structure. In addition, the sponsor may be well advised to spend extra money to foster Nash bargaining between management and chief physicians rather than the principal-agent alternative where management just makes a take-it-or-leave-it offer. The reason is that negotiation promises to avoid the accumulation of information rents, thus yielding a preferable outcome.

## Appendix

### Results in the case of full internal information

#### Hospital’s reporting with full internal information

The game is solved using backward induction. If the CP reports case severity always truthfully, i.e. $\hat {\theta }^{I} = \theta \;\forall \; \theta $, all she/he does at stage No. 4 is to decide on whether or not to treat the patient.

At stage No. 3 however, the CP decides on whether to accept M’s offer or to engage in bargaining. Assume that the CP opts for bargaining. In this case the paths of *t*^*I*^(*θ*) and $\hat {\theta }^{E}(\theta)$ are negotiated between the two players, given the contract {*t*^*E*^(*θ*),*q*^*E*^(*θ*)} offered by the sponsor to M at stage No. 1. Since both parameters must be determined *ex ante*, the players aim to maximize their share of expected surplus. The Nash bargaining outcome in terms of optimal payment and reporting is the solution to the maximization problem

19 $$\begin{array}{*{20}l}  \max_{t^{I}(\theta), \hat{\theta}^{E}(\theta)} \pi(\theta)& =\left(E\left[t^{E}\left(\hat{\theta}^{E}(\theta)\right) - t^{I}(\theta)\right] \right)^{1-w} \\&\quad \left(E \left[t^{I}(\theta) + \theta V\left(q^{E}(\hat{\theta}^{E}(\theta))\right)\right] \right)^{w} \end{array} $$

subject to CP’s and M’s (*ex-post*) participation constraints *U*≥0 and *P*≥0. To solve (19), note that surplus *S*(*θ*)=*P*(*θ*)+*U*(*θ*) is independent of payment *t*^*I*^(*θ*). Thus, *t*^*I*^(*θ*) can be used to achieve any arbitrary distribution of the surplus without affecting its total value. Since the Nash bargaining solution is Pareto-efficient (see e.g. [12] ch. 2), the optimal negotiated reporting strategy without internal asymmetry of information $\hat {\theta }^{E}(\theta)\;\forall \theta \in \Theta $ maximizes expected surplus,

20 $$  \max_{\hat{\theta}^{E}(\theta)} E[S(\theta)]= \int^{\overline{\theta}}_{\underline{\theta}}[ t^{E}(\hat{\theta}^{E}(\theta)) + \theta V(q^{E}(\hat{\theta}^{E}(\theta)))] f(\theta) d\theta,  $$

provided CP’s and M’s participation constraints do not bind. Assume this to be the case. Since (20) is additive in *θ* and *f*(*θ*), it suffices to maximize *ex-post* surplus *S*(*θ*) at a given value of *θ*, i.e.

21 $$  \max_{\hat{\theta}^{E}}S(\theta) = t^{E}(\hat{\theta}^{E}) + \theta V(q^{E}(\hat{\theta}^{E}))\;\; \forall \theta \in \Theta.  $$

The solution to (21) is denoted *efficient* reporting and the maximized surplus, *S*_*eff*_(*θ*).

While the reporting strategy $\hat {\theta }^{E}$ is used to maximize surplus, the optimal path of internal payment *t*^*I*^(*θ*) is used to distribute the surplus according to the CP’s relative bargaining power. Since both players are risk-neutral and assign the same weight to *t*^*I*^(*θ*), the choice of the path does not matter to them; rather they negotiate over the expected value *E*[*t*^*I*^(*θ*)]. Still assuming that both participation constraints do not bind, the first-order condition for the optimum of (19) w.r.t. *E*[*t*^*I*^(*θ*)] reads

22 $$  E[U(\theta)] = \frac{w}{1-w} E[P(\theta)]\;\; \text{or} \;\; E[U(\theta)] = w E[S(\theta)].  $$

Expression (22) states that through bargaining, the CP obtains an expected utility that equals expected joint surplus weighted by her/his relative bargaining power, leaving (1−*w*)*E*[*S*(*θ*)] as the expected profit for M.

As to the participation constraints, note that *S*(*θ*)=0 ∀*θ* and *S*(*θ*)>0 for at least one *θ* is necessary for both constraints not to bind in the optimum since *S*(*θ*)<0 would imply *P*(*θ*)<0 or *U*(*θ*)<0 or both. This condition is also sufficient, since it implies *E*[*S*(*θ*)]>0 and therefore ensures *P*(*θ*)≥0 and *U*(*θ*)≥0 for all *θ*. It follows that, given the sponsor’s payment system enables *S*(*θ*)=0 ∀*θ* and *S*(*θ*)>0 for at least one *θ*, the reporting strategy is efficient if the CP engages in bargaining.

At stage No. 2, M determines the initial offer $\{t^{I}(\theta), \hat {\theta }^{E}(\theta)\}$ for all *θ*, given the contract {*t*^*E*^(*θ*),*q*^*E*^(*θ*)} offered by the sponsor to M at stage No. 1. Again *t*^*I*^(*θ*) and $\hat {\theta }^{E}(\theta)$ must be determined *ex ante*, i.e. before the patient’s severity is established and reported. Thus M aims at maximizing expected profit

23 $$  E[P(\theta)] = E[t^{E}(\hat{\theta}^{E}(\theta))] - E[t^{I}(\theta)].  $$

or, with $U(\theta)=t^{I}(\theta) + \theta V(q^{E}(\hat {\theta }^{E}))$

24 $$  E[P(\theta)] = E[S(\theta)] - E[U(\theta)].  $$

subject to CP’s participation constraint. Further, M anticipates that the CP engages in bargaining at the next stage if this is to her/his of advantage. The contract must therefore also ensure that *E*[*U*(*θ*)]≥*wE*[*S*_*eff*_(*θ*)], in order to dissuade the CP from bargaining at the next stage. Since M wants to keep CP utility as low as possible, this inequality constraint binds in the optimum and M in fact aims at maximizing

25 $$  E[P(\theta)] = E[S] -wE[S_{eff}(\theta)].  $$

Because *wE*[*S*_*eff*_(*θ*)] is constant, maximizing (25) is equivalent to maximizing expected joint surplus. Therefore, the optimal reporting strategy is efficient as in the case of bargaining. It follows that M’s expected profit equals the share of efficient surplus *E*[*P*]=(1−*w*)*E*[*S*_*eff*_] that M would achieve with bargaining, causing *t*^*I*^(*θ*) to be the same as with bargaining and M to be indifferent between the two mechanisms.

#### Optimal payment system with full internal information

At stage No. 1, the sponsor designs the contract taking into account hospital behavior. By the revelation principle, the optimal allocation is achieved by a direct mechanism {*t*^*E*^(*θ*),*q*^*E*^(*θ*)} that induces truthful reporting, i.e. $\hat {\theta }^{E}(\theta)= \theta $. This means that the hospital’s incentive compatibility constraint

26 $$  \dot{S} (\theta) \equiv \frac{d S (\theta)}{d \theta}= V(q^{E}(\theta)) > 0  $$

must be satisfied. This condition is derived from applying the envelope theorem to the objective function (21) (see e.g. [9] ch. 1). Condition (26) states that for truthful reporting, the payment system {*t*^*E*^(*θ*),*q*^*E*^(*θ*)} must ensure that the joint surplus available to CP and M is increasing with case severity.

In designing the contract, the sponsor aims to maximize expected patient utility net of expenditure,

27 $$  E[W(\theta)] = \int^{\overline{\theta}}_{\underline{\theta}} \left [ \theta B(q^{E}(\theta)) - t^{E}(\theta)- q^{E}(\theta) \right ] f\theta d\theta,  $$

while considering hospital’s incentive compatibility constraint (26) as well as the participation constraint

28 $$  S(\theta)\geq 0 \; \forall \theta \in \Theta \; \text{and} \; S(\theta) > 0 \; \text{for at least one} \; \theta.  $$

Further

29 $$  \frac{d q^{E} (\theta)}{d \theta} \geq 0 \;\; \forall \; \theta  $$

must be satisfied to ensure that truthful reporting is globally optimal (for a proof, see again e.g. [9] ch. 1). For the moment, assume that condition (29) is satisfied. It will be verified below whether it holds in equilibrium (see expression (38)).

Since *S*(*θ*)=*t*^*E*^(*θ*)+*θV*(*q*^*E*^(*θ*)),

30 $$  \int^{\overline{\theta}}_{\underline{\theta}} \left[ t^{E}(\theta) \right] f\theta d\theta = \int^{\overline{\theta}}_{\underline{\theta}} \left[ \theta V(q^{E}(\theta))-S(\theta) \right] f\theta d\theta.  $$

Substituting (30) into (27) one obtains as the sponsor’s maximization problem,

31 $$ \begin{aligned} &\max_{q^{E}(\theta), S(\theta)} E[W(\theta)]\! =\! \int^{\overline{\theta}}_{\underline{\theta}} \left [ \theta B(q^{E}(\theta)) \!+ \theta V(q^{E}(\theta)) - q^{E}(\theta) - S(\theta) \right ] f\theta d\theta \\  & s.t. \\ & \dot{S}(\theta) = V(q^{E}(\theta))>0 \\ &S(\theta)\geq 0 \; \forall \theta \in \Theta \; \text{and} \; S(\theta) > 0 \; \text{for at least one} \; \theta. \end{aligned}  $$

To solve this, note that the incentive compatibility constraint requires surplus *S*(*θ*) to be strictly increasing in *θ*. Since the sponsor seeks to keep the surplus as small as possible to reduce expenditure, the participation constraint binds at the lowest value of severity,

32 $$  S(\underline{\theta}) = 0.  $$

The incentive condition (26) together with (32) determines surplus,

33 $$  S(\theta) = \int^{\theta}_{\underline{\theta}} V(q^{E}(\tilde{\theta})) d\tilde{\theta}.  $$

Thus expected surplus can be written as

34 $$  ES(\theta)] = \int^{\overline{\theta}}_{\underline{\theta}} \int^{\theta}_{\underline{\theta}} V(q^{E}(\tilde{\theta}))d\tilde{\theta} f(\theta) d\theta.  $$

Integration by parts results in

35 $$  E(S(\theta)) = \int^{\overline{\theta}}_{\underline{\theta}} h(\theta) V(q^{E}(\theta)) f(\theta) d\theta,  $$

with $h(\theta) \equiv \frac {1- F(\theta)}{f(\theta)}$ denoting the inverse hazard rate. Inserting (35) into the sponsor’s objective function, one can rewrite (31) as the unrestricted maximization problem

36 $$\begin{array}{*{20}l}  \max_{q^{E}(\theta)}E[W(\theta)] &= \int^{\overline{\theta}}_{\underline{\theta}}[\theta B(q^{E} (\theta)) + \theta V(q^{E} (\theta))\\&\quad - q^{E} (\theta) - h(\theta)V(q^{E} (\theta)) ]f(\theta) d(\theta) \end{array} $$

Assuming the integrand in (36) to be concave and continuously differentiable, point-wise maximization can be applied, resulting in the first-order condition with respect to *q*^*E*^(*θ*),

37 $$  \theta B_{q} (q^{E}(\theta)) + (\theta -h(\theta)) V_{q}(q^{E}(\theta)) = 1,  $$

where the subscript denotes the first derivative w.r.t. *q*. This establishes Eq. (7) in the text.

The optimal path *q*^*E*^(*θ*) satisfies the necessary condition (29). Differentiating (37) with respect to *θ* yields

38 $$  \frac{dq^{E}(\theta)}{d\theta}=-\frac{B_{q} (q^{E}(\theta))+(1-h'(\theta))V_{q} (q^{E}(\theta))}{\theta B_{qq} (q^{E}(\theta)) + (\theta -h(\theta)) V_{qq}(q^{E}(\theta))}>0;  $$

the sign follows from *h*^′^(*θ*)<0.

### Efficient reporting with bargaining

The maximization problem with bargaining and internal asymmetry of information reads

39 $$ \begin{aligned} &\pi(\theta) = \left(E\left[t^{E}(\hat{\theta}^{E}(\theta)) - t^{I}(\theta)\right] \right)^{1-w} \left(E\left[t^{I}(\theta) + \theta V(q^{E}[\hat{\theta}^{E}(\theta)])\right] \right)^{w}\\  \end{aligned}  $$

s.t.

40 $$\begin{array}{*{20}l}  &&\dot{U}(\theta) = V(q^{E}(\hat{\theta}^{E}(\theta))) >0 \;\forall \theta \in \Theta, \end{array} $$

41 $$\begin{array}{*{20}l}  && U(\underline{\theta}) \geq 0, \end{array} $$

42 $$\begin{array}{*{20}l}  && P(\theta) \geq 0 \; \forall \; \theta \in \Theta. \end{array} $$

Notice that if neither participation constraint (41) nor (42) is binding, the incentive compatibility constraint (40) does not constrain the maximization either since it can be attained by structuring the path of the internal transfer *t*^*I*^(*θ*) ∀*θ*∈*Θ* accordingly. This path may be determined such that condition (40) is satisfied while leaving the joint surplus unaffected. This allows the optimal reporting strategy $\hat {\theta }^{E}(\theta)$ to be chosen such that it maximizes joint surplus.

In fact, both participation constraints do not bind, given the payment system designed by the sponsor ensures that the joint surplus exceeds CP’s information rent for every level of severity, i.e. *S*_*eff*_(*θ*)>*R*_*eff*_(*θ*) ∀*θ*:

#### **Proposition 1**

If the sponsor employs a payment system that yields *S*_*eff*_(*θ*)=*R*_*eff*_(*θ*)+*ε* ∀ *θ* with *ε*>0, there uniquely exists a $\bar {w} = \frac {E[R_{eff}]}{E[R_{eff}]+ \epsilon }$ such that the unrestricted bargaining solution implies *U*(*θ*)≥0 ∀ *θ* if $w \geq \bar {w}$ while *P*(*θ*)>0 ∀ *θ*.

*Proof* Writing the CP’s ex-post utility with the bargaining solution as the sum of her/his information rent and a constant *k*, one has

43 $$  U(\theta) = R(\theta) + k.  $$

with $R(\theta) = \int ^{\theta }_{\underline {\theta }} V(q^{E}(\hat {\theta }^{E}(\tilde {\theta }))) d\tilde {\theta }$. Since *k* is the level of utility obtained by CP for the lowest severity level $\theta =\underline {\theta }$, it follows that CP’s participation constraint is not binding for all *θ* if the solution yields *k*≥0.

Recall that the unrestricted bargaining solution is characterized by an efficient reporting strategy that maximizes joint surplus and an expected utility for the CP that equals *wE*[*S*_*eff*_] (see Eq. (22)). Using (43), one therefore obtains

44 $$ wE[S_{eff}] = E[R_{eff}] + k.  $$

It follows that

45 $$ k = wE[S_{eff}] - E[R_{eff}] \geq 0 \; \leftrightarrow \; w \geq \bar{w} := \frac{E[R_{eff}]}{E[S_{eff}]},  $$

that is, with efficient reporting *k*≥0 holds if $w \geq \bar {w}$, which implies that the bargaining solution in fact is unrestricted if $w \geq \bar {w}$. Further, provided the sponsor employs a payment system {*t*^*E*^(*θ*),*q*^*E*^(*θ*)} that yields *S*_*eff*_(*θ*)=*R*_*eff*_(*θ*)+*ε* with $\epsilon > 0, \bar {w}$ lies between 0 and 1 because $\frac {E[R_{eff}]}{E[S_{eff}]}=\frac {E[R_{eff}]}{E[R_{eff}]+ \epsilon }\; \in \; (0,1)$. Also, since $\frac {d\bar {w}}{d \epsilon } < 0, \bar {w}$ is unique.

On the other hand, M’s participation constraint also is not binding, since *S*_*eff*_(*θ*)=*R*_*eff*_(*θ*)+*ε* ∀ *θ* implies *E*[*S*_*eff*_]=*E*[*R*_*eff*_](*θ*)+*ε*. Because *E*[*S*_*eff*_]>*wE*[*S*_*eff*_] from (44) one has *ε*>*k*. Since *P*(*θ*)=*S*(*θ*)−*R*(*θ*)−*k*=*ε*−*k*, it follows that *P*(*θ*)>0 ∀ *θ*.

From Proposition 1 it follows that if CP’s bargaining power exceeds $\bar {w}$, the bargaining solution indeed maximizes surplus even if internal asymmetry of information is present.

### Optimal reporting with internal asymmetric information in the case of the PA mechanism

M seeks to maximize expected profit

46 $$  \max_{\hat{\theta}^{E}(\theta), t^{E}(\theta)}E[P(\theta)] = E[ t^{E}(\hat{\theta}^{E}(\theta))-t^{I}(\theta)].  $$

subject to CP’s participation constraint and CP’s incentive compatibility constraint. With *U*(*θ*)=*θV*(*q*)+*t*^*I*^(*θ*), M’s maximization problem thus can be written as

47 $$\begin{array}{*{20}l}  \max_{\hat{\theta}^{E}(\theta), U(\theta)}E[P(\theta)] &\,=\, \int^{\overline{\theta}}_{\underline{\theta}}\![ \!t^{E}(\hat{\theta}^{E}(\theta))\! +\! \theta V(q^{E}(\hat{\theta}^{E}(\theta)))] f(\theta) d\theta \\&\quad- \int^{\overline{\theta}}_{\underline{\theta}} U(\theta) f(\theta) d\theta  \\ & \\ &s.t.  \\ &\dot{U}(\theta) \!= V(q^{E}(\hat{\theta}^{E}(\theta))), \; U(\theta) \geq 0 \;\forall \theta \in \Theta.  \end{array} $$

Further, for truthful reporting to be globally optimal, M’s reporting strategy must ensure that

48 $$  \frac{d q^{E}(\hat{\theta}^{E}(\theta))}{d \theta} \geq 0 \;\; \forall \; \theta  $$

For the moment, assume that condition (48) is satisfied. This will be verified below in Appendix 2, expression (67).

CP’s incentive compatibility constraint implies

49 $$  U(\theta) = \int^{\theta}_{\underline{\theta}}V(q^{E}(\hat{\theta}^{E}(\tilde{\theta}))) d\tilde{\theta}+ a,  $$

where *a* is a constant. Hence, CP’s expected utility can be written as

50 $$  EU(\theta)] = \int^{\overline{\theta}}_{\underline{\theta}} \int^{\theta}_{\underline{\theta}} V(q^{E}(\hat{\theta}^{E}(\tilde{\theta})))d\tilde{\theta} f(\theta) d\theta + a.  $$

Integration by parts results in

51 $$  E[U(\theta)] = \int^{\overline{\theta}}_{\underline{\theta}} h(\theta)V(q^{E}(\hat{\theta}^{E}(\theta))) f(\theta) d\theta + a,  $$

with $h(\theta) \equiv \frac {1- F(\theta)}{f(\theta)}$ again denoting the inverse hazard rate.

M dislikes leaving any surplus to the CP, whose incentive compatibility constraint however demands that utility increase with case severity. M therefore sets $U(\underline {\theta })=0$, implying *a*=0. Hence the maximization problem of M, using (51), and *a*=0, reduces to

52 $$\begin{array}{*{20}l}  \max_{\hat{\theta}^{E}(\theta)}E[P(\theta)] &= \int^{\overline{\theta}}_{\underline{\theta}}[ t^{E}(\hat{\theta}^{E}(\theta)) + \theta V(q^{E}(\hat{\theta}^{E}(\theta)) \\&\quad- h(\theta) V(q^{E}(\hat{\theta}^{E}(\theta)))] f(\theta) d\theta. \end{array} $$

Equivalently, once again relying on point wise maximization, one has

53 $$ \begin{aligned}  \max_{\hat{\theta}^{E}(\theta)}\tilde{P}(\theta) = t^{E}(\hat{\theta}^{E}) + \theta V(q^{E}(\hat{\theta}^{E}(\theta))) - h(\theta) V(q^{E}(\hat{\theta}^{E}(\theta))) \;\forall \theta \in \Theta. \end{aligned}  $$

This is expression (14) in the text.

### Equilibrium mechanism with internal asymmetry of information

At stage No. 2, assume that M offers the contract defined in “The solution to the PA mechanism” section. Since this contract guarantees the CP an expected information rent *E*[*R*_*PA*_]>0, the bargaining alternative comes about only if CP’s bargaining power *w* is high enough such that her/his expected share of (efficient) surplus exceeds *E*[*R*_*PA*_].

Clearly this is the case if $w \geq \bar {w} = \frac {E[R_{eff}]}{E[S_{eff}]}$, since *E*[*R*_*eff*_]>*E*[*R*_*PA*_], i.e. CP’s information rent with efficient reporting exceeds the information rent given inefficient reporting in the PA setting. On the other hand, the CP’s bargaining power may be so low that even the bargained share of the *efficient* surplus falls short of the expected information rent in the inefficient PA setting. Denote this level with $\underline {w} := \frac {E[R_{PA}]}{E[S_{eff}]}$; therefore if $w < \underline {w}$, the PA mechanism would prevail.

Further, given $w < \bar {w}=\frac {E[R_{eff}]}{E[S_{eff}]}$, the CP’s participation constraint becomes relevant in the maximization problem of the bargaining solution of (11), causing the bargained reporting strategy to be distorted away from the efficient strategy. Obviously, this distortion increases and surplus decreases as *w* decreases, making the constraint more binding. Consequently, CP’s share of surplus strictly decreases as *w* decreases. Therefore there is a threshold level of bargaining power $w \in [\underline {w}, \bar {w}]$ for which the CP is indifferent between bargaining and accepting the PA alternative. If CP’s bargaining power is above this threshold, the CP engages in bargaining, if it is below, she/he accepts the PA mechanism.

The crucial insight, however, is that any level of bargaining power $w < \bar {w}$ precludes efficient reporting. Therefore, a formal derivation of this threshold and hospital behavior associated with it does not seem worthwhile. Instead the CP is assumed to engage in bargaining iff $w \geq \bar {w}$. Therefore M offers the contract defined in “The solution to the PA mechanism” section at stage No. 2 if $w < \bar {w}$ since this contract implies the optimal trade-off between efficiency and rent elicitation. If however $w \geq \bar {w}$ holds, M could dissuade the CP from bargaining by paying a sufficiently high lump sum in addition to the PA contract. However, this would not be optimal for M. If $w \geq \bar {w}$, the bargaining solution yields an expected utility of *wE*[*S*_*eff*_] for the CP, while with the PA solution defined in (“The solution to the PA mechanism”) section her/his expected utility equals the expected information rent *E*[*R*_*PA*_]. Therefore, to dissuade the CP from bargaining and to make her/him accept the PA solution, M would have to pay a lump sum in addition to the PA contract amounting to

54 $$  b = wE[S_{eff}] - E[R_{PA}].  $$

With (54), the net profit for M from avoiding a bargaining solution amounts to

55 $$ \begin{aligned} \Delta E[P] &= E[P_{PA}]- b -E[P_{eff}]\\  & =\left(E[S_{PA}] - E[R_{PA}] \right) \,-\, \left(wE[S_{eff}] - E[R_{PA}] \right) \,-\, \left((1-w)E[S_{eff}] \right) \\ & = E[S_{PA}] -E[S_{eff}] < 0. \end{aligned}  $$

The negative sign follows from the fact that the surplus with the inefficient PA solution is strictly below the efficient surplus. Therefore it is better for M to accept the bargaining solution.

### Optimal allocation with internal asymmetry of information and PA setting

At stage No. 1, the sponsor aims to maximize expected patient utility net of expenditure,

56 $$  E[W(\theta)] = \int^{\overline{\theta}}_{\underline{\theta}} \left [ \theta B(q^{E}(\theta)) - t^{E}(\theta)- q^{E}(\theta) \right ] f\theta d\theta  $$

in determining the optimal payment system. Since *S*(*θ*)=*t*^*E*^(*θ*)+*θV*(*q*^*E*^(*θ*)),

57 $$  \int^{\overline{\theta}}_{\underline{\theta}} \left[ t^{E}(\theta) \right] f(\theta) d\theta = \int^{\overline{\theta}}_{\underline{\theta}} \left[ \theta V(q^{E}(\theta))-S(\theta) \right] f(\theta) d\theta.  $$

After substitution of (57) into (56), the sponsor’s objective function can be written as

58 $$ \begin{aligned} E[W(\theta)] = \int^{\overline{\theta}}_{\underline{\theta}} \left [ \theta B(q^{E}(\theta)) + \theta V(q^{E}(\theta)) - q^{E}(\theta) - S(\theta) \right ] f(\theta) d\theta.\\ \end{aligned}  $$

The sponsor now needs to consider the incentive compatibility constraint

59 $$  \dot{\tilde{P}}(\theta) = (1 - h'(\theta)) V(q^{E}(\theta)) > 0.  $$

Furthermore

60 $$  \dot{q}^{E}(\theta)\geq 0 \;\; \forall \; \theta  $$

must be satisfied to ensure that truthful reporting is globally optimal. For the moment, assume that condition (60) is satisfied. This will be verified below in expression (67).

Integrating (59) yields

61 $$  \tilde{P}(\theta) = \int^{\theta}_{\underline{\theta}} (1 - h'(\tilde{\theta})) V(q^{E}(\tilde{\theta})) d\tilde{\theta} + g  $$

with *g* as a constant. With (61), expected profit can be written as

62 $$ E[P(\theta)]= \int^{\overline{\theta}}_{\underline{\theta}} \int^{\theta}_{\underline{\theta}} (1 - h'(\tilde{\theta})) V(q^{E}(\tilde{\theta})) d\tilde{\theta} f(\theta) d\theta + g.  $$

Adding CP’s expected utility defined by expression (50) (where *a*=0), the expected surplus writes

63 $$  E[S(\theta)] = \int^{\overline{\theta}}_{\underline{\theta}} \int^{\theta}_{\underline{\theta}} (2 - h'(\tilde{\theta})) V(q^{E}(\tilde{\theta})) d\tilde{\theta} f(\theta) d\theta + g.  $$

Applying integration by parts, one obtains

64 $$  E[S(\theta)] = \int^{\overline{\theta}}_{\underline{\theta}} h(\theta)(2 - h'(\theta)) V(q^{E}(\theta))f(\theta) d\theta + g  $$

Inserting (64) into (58), the sponsor’s maximization problem can be formulated as

65 $$\begin{array}{*{20}l}  \max_{q^{E}(\theta),g} E[W(\theta)] &= \int^{\overline{\theta}}_{\underline{\theta}} \theta B(q^{E}(\theta)) + \theta V(q^{E}(\theta)) - q^{E}(\theta)\\&\quad - h(\theta)(2 - h'(\theta) V(q^{E}(\theta)) f(\theta) d\theta - g \end{array} $$

subject to M’s participation constraint.

Point wise maximization of (65) yields the first-order condition

66 $$  \theta B_{q}(q^{E}(\theta)) +\left(\theta - h(\theta)(2 - h'(\theta)\right)V_{q}(q^{E} (\theta)) = 1.  $$

This is equation (16) in the text. Further,

67 $$ \begin{aligned}  \frac{dq^{E}(\theta)}{d\theta}\,=\,-\!\frac{B_{q} (q^{E}(\theta))+\left(1\,-\,2h'(\theta)\,+\,h'(\theta)\,+\,h(\theta)h^{\prime\prime}(\theta) \right) V_{q} (q^{E}(\theta))}{\theta B_{qq}(q^{E}(\theta)) \,+\,\left(\theta - h(\theta)(2 - h'(\theta)\right)V_{qq}(q^{E}(\theta))}\!>\!0. \end{aligned}  $$

This is positive because of *h*(*θ*)>0,*h*^′^(*θ*)<0) and *h*^′′^(*θ*)≥0.

Since M’s profit as well as CP’s utility must be increasing in *θ*, it follows that

68 $$ \dot{S}(\theta)>0.  $$

With the PA mechanism, CP attains a reservation utility of zero for treating a patient with minimum severity $\underline {\theta }$. Thus the participation constraint of M can be written in terms of ex-post surplus as

69 $$  S(\underline{\theta}) \geq 0.  $$

From (64) follows $S(\underline {\theta })=g$. The sponsor optimally sets *g*=0, implying

70 $$ S(\underline{\theta}) = 0.  $$

## Data Availability

Not applicable.

## Abbreviations

*B*: Unweighted patient’s benefit
CP: Chief physician
_*eff*_: Subscript denoting the case of efficient hospital’s reporting
*h*(*θ*): Inverse hazard rate
M: Management
*P*: Objective function of M
*PA*: Principal agent
*q*: Service quantity / treatment cost
*q*^*E*^: External cost target
*q*^*I*^: Internal cost target
*R*: CP’s information rent
*S*: Joint surplus of M and CP
*t*^*I*^: Internal transfer
*t*^*E*^: External transfer
*θ*: Case severity
*θ*^*E*^: Hospital’s external report of case severity
*θ*^*I*^: CP’s internal report of case severity
*U*: Objective function of CP
*V*: CP’s unweighted valuation of treatment
*W*: Objective function of the sponsor
*w*: CP’s relative bargaining power
$\bar{w}$: Minimum of CP’s bargaining power necessary for the CP to engage in bargaining
$\bar{w}^{*}$: Minimum of CP’s bargaining power necessary for the sponsor to buy efficiency

## Footnotes

1 This implies that treatment cost is independent of case severity. This is in contrast to the common assumption that treatment cost rises with the severity of illness. In fact, treatment cost rises with the resources used for treatment. A positive correlation between cost and severity therefore results only if the resources used for treatment increase with case severity, which is not given but depends on the decision of the physician.

2 There may be a saturation quantity beyond which the negative effect of extra treatment outweighs the positive one. However, any equilibrium is assumed to be associated with a quantity below this level.

3 This participation constraint is ex-post, since it prevents the CP from dumping individual cases after observing severity. An ex-ante participation constraint would state that the CP needs to accept the complete contract, in which case the next-best alternative would be to leave the hospital in favor of another healthcare facility. This ex-ante participation constraint is satisfied along with the ex-post constraint.

4 This assumption is made to simplify the analysis and not to deny the importance of social and altruistic preferences on the part of hospital managers.

5 As in the case of the CP, M’s ex-ante participation constraint is satisfied along the ex-post constraint.

6 For a discussion see [13] ch. 4.

7 Given space constraints, the analysis of the case with full internal information is only sketched in the main text. The complete analysis is provided in Appendix A.

8 Alternatively, the CP might honor the agreement even if a false report would yield a higher utility. In this case, the bargaining solution need not satisfy the incentive compatibility constraint (9). However, here it is assumed that the CP behaves non-cooperatively, exploiting her/his informational advantage at stage No. 4 despite her/his prior involvement in decision making at stage No. 3.
